# Thoracic Splenosis - A necessary differential diagnosis for pleural based nodules with history of thoracoabdominal trauma

**DOI:** 10.12669/pjms.38.3.4563

**Published:** 2022

**Authors:** Faisal Mehmood, Muhammad Murad Murtaza, Shehrbano Ali, Amna Ashraf

**Affiliations:** 1 Dr. Faisal Mehmood, FCPS (Medicine), FCPS (Medical Oncology), MCPS, OJT (Acute Medicine), OJT (Medical Oncology). Head of Department, Department of Oncology, CMH Lahore, Pakistan; 2 Muhammad Murad Murtaza, MBBS Student. CMH Lahore Medical College, Lahore, Pakistan; 3 Shehrbano Ali, MBBS Student. CMH Lahore Medical College, Lahore, Pakistan; 4 Dr. Amna Ashraf, FCPS (Medicine). Department of Medicine, CMH Lahore, Pakistan

**Keywords:** thoracic splenosis, accessory spleen, trauma, malignancy, splenectomy, spleen

## Abstract

Thoracic Splenosis (TS) is a rare medical condition, where there is auto-transplantation of the splenic tissue in the thoracic cavity, often leading to pleural based nodules. Our patient is the first ever case of this condition in Pakistan, and underwent extensive diagnostic procedures as well as medical treatments, before receiving the diagnosis of TS. He underwent HRCT for chronic cough that revealed pleural and mediastinal nodules. This coupled with a vague mass in the testes led to the provisional diagnosis of metastasized testicular tumour, and later a diagnosis of pulmonary tuberculosis was made. However, eventually a ^99m^Tc denatured red blood cell scan confirmed the diagnosis of TS. TS is a benign condition, whereas other causes of pleural nodules are relatively malignant, hence its diagnosis is essential in ruling out malignancies. Among the multiple invasive and non-invasive diagnostic modalities, the gold standard remains ^99m^Tc denatured red blood cell scan, which is a sensitive test that provides an accurate diagnosis and bars the need of multiple invasive procedures.

## INTRODUCTION

Thoracic Splenosis is a rare medical condition, with less than 100 cases reported in the literature thus far. It is defined as auto-transplantation of the splenic tissue in the thoracic cavity. The condition is usually a result of joint traumatic injury to the abdomen and diaphragm,^1^ and very rarely is thought to have a congenital origin as well.^2^ The condition usually remains asymptomatic and is only discovered as an incidental finding, however its diagnosis is necessary owing to the range of differentials associated with it, including malignant conditions.

Our case report describes one such patient of thoracic splenosis, and is the first case of this condition to be reported in Pakistan.

## CASE REPORT

Our patient has a history of gunshot wound at the age of 15 years. The bullet entered from the right side following a transverse trajectory to the left and then upwards into the thorax. The course of the bullet resulted in liver damage, entry and exit wound in the stomach, splenic rupture, rupture and herniation of diaphragm, and eventually exit of the bullet from the left lung causing hemopneumothorax. As a result, the patient underwent chest intubation and splenectomy. Owing to the splenectomy, the patient experienced recurrent infections in the following years, despite receiving pneumococcal, meningococcal, and H influenza vaccination. Regular blood tests also revealed high platelet count and Howell-Jolly Bodies were seen on the peripheral blood smear. Over next 10 years, however, the infections gradually decreased and the routine CBC showed normal platelet count.

At the age of 33 years, the patient then started experiencing chronic cough for which investigations were done. The HRCT revealed mild central bronchiectasis, along with one soft tissue nodule in left mediastinum adjacent to cardiac apex and multiple other pleural based nodules on left side. No attention was paid to the nodules and the symptoms resolved spontaneously.

Two years later, the patient again complained of non-resolving cough, along with approximately four kilograms of weight loss. A repeat CT was done and the nodules were observed again with an approximately 10% increase in size; the mediastinal nodule being the largest and measuring 2.1x1.5cm. This time, further investigations were done with regards to these nodules, including an ultrasound of whole body, followed by CT scan of neck, abdomen and pelvis. A faint doubtful hypo-echoic lesion was observed in left testes, and a provisional diagnosis of testicular tumour with lung metastasis was formed. On receiving this diagnosis, the patient started experiencing severe anxiety, and so was prescribed Escitalopram 10 mg OD as well as Propranolol 10 mg TDS.

Even though the germ cell tumour markers of AFP and beta-HCG were within normal range, a thoraco-oncology multidisciplinary team discussion reached the consensus for carrying out radical orchidectomy for the testicular mass. Histopathology of the sample later showed normal tissue with no evidence of malignancy. This was then followed by a bronchoscopy that revealed chronic non-specific inflammation, which led to a differential diagnosis of pulmonary tuberculosis, for which the patient was started on anti-tuberculosis treatment. After six months of treatment, a repeat CT was done, which showed no regression in nodule size.

The patient continued to remain asymptomatic, and went to UK where his case was reviewed at The New Queen Elizabeth Hospital Birmingham, and a denatured red cell sequestration study (^99m^Tc denatured red blood cell scan) was carried out. The study revealed significant red cell accumulation in the nodules consistent with splenunculi, and thus confirmed the diagnosis of thoracic splenosis in light of current findings and history of traumatic injury. Informed consent was taken from the patient to publish these findings.

**Fig.1:**
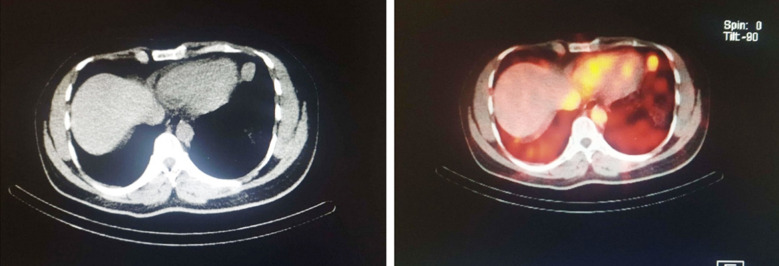
HRCT and 99m-Tc DRBC scan showing splenic nodule measuring 2.1x1.5cm in left mediastinum adjacent to cardiac apex.

**Fig.2:**
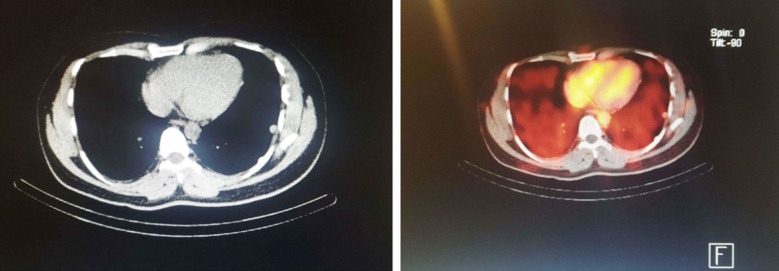
HRCT and 99m-Tc DRBC scan showing pleural based splenic nodules that show uptake of denatured RBCs.

## DISCUSSION

Thoracic splenosis (TS) was first discovered in an autopsy of a 25-year old male in 1896,^3^ and later in six autopsies reported by Shaw and Shafi in 1937.^4^ Since then, less than hundred cases have been reported, with its incidence being a mere 18%,^5^ most likely owing to the asymptomatic nature of the disease causing it to remain undiagnosed. The disease is seen to have a male predominance, and gradually develops on average 18.8 years after traumatic thoracoabdominal injury,^6^ mostly due to gunshots or road traffic accidents. In our patient too, it was first observed 18 years after the gunshot injury.

TS appears to have an advantageous function in splenectomised individuals who are immunocompromised and at an increased risk of severe infections owing to the absence of a spleen. In such cases, the auto-transplanted splenic tissue serves as an accessory spleen, and can thus decrease the risk of infections.^7^ However, the degree of immunoprotection provided by this accessory spleen remains unclear and doubtful.^8^

Confirming the diagnosis of TS is essential as pleural based nodules enlist multiple differential diagnosis, including but not limited to metastatic tumor, lymphoma, post traumatic scarring, malignant mesothelioma, infectious diseases, empyema, hamartoma, schwannoma, rheumatological lesions, atelectasis, invasive thymoma and sclerosing hemangioma.^6^,^9^ It is to be noted that many of these conditions are malignant and quite serious in nature, while TS is relatively benign. Lack of awareness of the condition can thus lead to excessive and unnecessary medical and surgical investigations and interventions, as well as become a source of psychological trauma, as seen in our patient who had to take anxiolytics after receiving the incorrect diagnosis of metastasized testicular tumor.

There are multiple different modalities for reaching a diagnosis of TS. Pleural based nodules on the left side of chest picked up on X-ray, CT scan or MRI, in the presence of thoracoabdominal injury especially with splenic rupture and diaphragmatic herniation, should be one of the very first indications of TS. Furthermore, the absence of Howell-Jolly bodies, Heinz bodies and siderocytes on the peripheral film, as well as a normal platelet count in splenectomised individuals should also be an indication of TS, as such individuals commonly present with increased platelet count and the aforementioned findings on smear.^8^ At times, the diagnosis may even require a thoracotomy, excision of suspected mass, CT-guided biopsy, or fine needle aspiration cytology in order to rule out the presence of a malignancy.

One very important non-invasive diagnostic modality for TS is that of nuclear imaging. Nuclear imaging techniques include SPECT or scintigraphy labelled with ^99m^Technetium (Tc) sulfur colloid, indium 111-labeled platelet, ^99m^Tc denatured red blood cell, or ^99m^Tc white blood cell. Among these, the most specific is the ^99m^Tc denatured red blood cell scan where the uptake of the denatured RBCs can be seen even in those splenunculi that could not be identified on CT imaging, making this diagnostic method more effective than all others.^9^,^10^

Once the diagnosis of TS is confirmed, its management is merely symptomatic if needed. Removal of the accessory splenic tissue via surgery is unnecessary as the nodules are unlikely to cause any problem besides mass effect depending upon their location. Leaving the splenic tissue in the body will also serve the benefit of combatting infections in the absence of a spleen.

## CONCLUSION

In conclusion, thoracic splenosis is an important diagnosis to be considered in cases of asymptomatic pleural based nodules and history of joint traumatic injury to the thorax and abdomen, and should not be mistaken for malignant conditions that may resemble it on imaging. The most effective diagnostic technique for TS remains to be ^99m^Tc denatured red blood cell scan, which can diagnose even very small splenic nodules, and thus bar the need for other diagnostic or invasive procedures.

### Authors Contribution:

**FM:** Acquisition of data and reviewed the manuscript.

**MMM & SA:** Conception and manuscript writing.

**AA:** Acquisition of data, review and final approval of manuscript.
